# Peripheral hearing loss at age 70 predicts brain atrophy and associated cognitive change

**DOI:** 10.1136/jnnp-2023-333101

**Published:** 2024-04-03

**Authors:** Thomas D Parker, Chris Hardy, Sarah Keuss, William Coath, David M Cash, Kirsty Lu, Jennifer M Nicholas, Sarah-Naomi James, Carole Sudre, Sebastian Crutch, Doris-Eva Bamiou, Jason D Warren, Nick C Fox, Marcus Richards, Jonathan M Schott

**Affiliations:** 1 Department of Brain Sciences, Imperial College London, London, UK; 2 The Dementia Research Centre, Department of Neurodegenerative Disease, University College London, London, UK; 3 UK Dementia Research Institute Centre for Care Research and Technology, Imperial College London, London, UK; 4 UK Dementia Research Institute at UCL, University College London, London, UK; 5 Department of Medical Statistics, London School of Hygiene and Tropical Medicine, London, UK; 6 MRC Unit for Lifelong Health and Ageing, University College London, London, UK; 7 Centre for Medical Image Computing, University College London, London, UK; 8 School of Biomedical Engineering and Imaging Sciences, King's College London, London, UK; 9 UCL Ear Institute and UCLH Biomedical Research Centre, National Institute for Health Research, University College London, London, UK

**Keywords:** ALZHEIMER'S DISEASE, AMYLOID, CEREBROVASCULAR DISEASE, COGNITIVE NEUROPSYCHOLOGY, DEMENTIA

## Abstract

**Background:**

Hearing loss has been proposed as a modifiable risk factor for dementia. However, the relationship between hearing, neurodegeneration, and cognitive change, and the extent to which pathological processes such as Alzheimer’s disease and cerebrovascular disease influence these relationships, is unclear.

**Methods:**

Data from 287 adults born in the same week of 1946 who underwent baseline pure tone audiometry (mean age=70.6 years) and two time point cognitive assessment/multimodal brain imaging (mean interval 2.4 years) were analysed. Hearing impairment at baseline was defined as a pure tone average of greater than 25 decibels in the best hearing ear. Rates of change for whole brain, hippocampal and ventricle volume were estimated from structural MRI using the Boundary Shift Integral. Cognition was assessed using the Pre-clinical Alzheimer’s Cognitive Composite. Regression models were performed to evaluate how baseline hearing impairment associated with subsequent brain atrophy and cognitive decline after adjustment for a range of confounders including baseline β-amyloid deposition and white matter hyperintensity volume.

**Results:**

111 out of 287 participants had hearing impairment. Compared with those with preserved hearing, hearing impaired individuals had faster rates of whole brain atrophy, and worse hearing (higher pure tone average) predicted faster rates of hippocampal atrophy. In participants with hearing impairment, faster rates of whole brain atrophy predicted greater cognitive change. All observed relationships were independent of β-amyloid deposition and white matter hyperintensity volume.

**Conclusions:**

Hearing loss may influence dementia risk via pathways distinct from those typically implicated in Alzheimer’s and cerebrovascular disease in cognitively unimpaired older adults.

## Introduction

Hearing loss has been proposed as a risk factor for dementia.^1–4^ However, the mechanisms by which hearing loss may influence neurodegeneration and cognitive decline are uncertain. In cross-sectional analyses, we have previously shown that peripheral hearing ability measured with pure tone audiometry did not predict β-amyloid (Aβ) deposition, white matter hyperintensity volume (WMHV) or Alzheimer’s disease-pattern neurodegeneration in adults aged approximately 70 years born in the same week of 1946.^5^ Building on this work, we used longitudinal data from the same cohort to investigate how hearing ability, Aβ-deposition and WMHV influence subsequent change in cognitive performance and brain atrophy.

## Methods

### Participants

We included data from 287 participants born in the same week of 1946 who underwent two time point cognitive assessment/multimodal brain imaging (mean age at baseline 70.5 years, mean interval between assessments 2.4 years) as part of Insight-46, a substudy of the MRC National Survey of Health and Development.^6^

Exclusions from the original Insight 46 sample (n=502) included no baseline imaging (n=31); baseline imaging quality control failure (n=15); pre-existing diagnosis of mild cognitive impairment, dementia or major neurological disorder (n=48); confounding otological pathology (n=16); hearing testing equipment unavailable (n=19); missing *APOE* genotype (n=2); missing socioeconomic position data (n=3) and no longitudinal follow-up visit data (n=81).

### Hearing assessment

Baseline hearing assessment included obtaining audiometric thresholds for each ear at 0.5, 1, 2 and 4 kHz using calibrated Maico-MA-25 audiometers with sound-excluding TDH-49 earphones in audiocups. Pure-tone averages (PTAs) in the better hearing ear were calculated using thresholds for 0.5, 1, 2 and 4 kHz.^5^ Hearing impairment was defined as a PTA greater or equal to 25 dB HL.

### Imaging analysis

Florbetapir PET and MRI data were acquired on a Single Siemens Biograph 3-Tesla PET/MRI scanner. Aβ-status (negative/positive) at baseline was determined using previously published methodology.^5 7^ WMHV was estimated using BaMoS.^8^ Total intracranial volume (TIV) was calculated using Statistical Parametric Mapping 12. Changes in whole-brain, ventricular and hippocampal volume were calculated from baseline and repeat 3D T1-weighted MRI with the boundary shift integral (BSI).^7 9^

### Cognitive testing

Cognition was assessed using an adapted version of the Preclinical Alzheimer’s Cognitive Composite (PACC), composed of the following tests: Mini-Mental State Examination, Logical Memory IIa from the Wechsler Memory Scale-Revised, Digit-Symbol Substitution test from the Wechsler Adult Intelligence Scale-Revised and the 12-item Face-Name test.^10^

### Statistical analysis

Wilcoxon rank sum tests, t-tests and Fisher’s exact test were used to assess unadjusted associations between demographic variables and hearing impairment (table 1).

**Table 1:** Relationship between peripheral hearing ability and baseline demographics

	Normal peripheral hearing (n=176)	Peripheral hearing impaired (n=111)	P value	Association with PTA
Age at baseline, years, mean (SD)	70.5 (0.6)	70.5 (0.6)	0.39*	r=0.06 (p=0.3)†
Female, n (%)	91 (51.7)	47 (42.3)	0.15‡	p=0.25*
TIV, mL, mean (SD)	1430 (135)	1445 (128)	0.32¶	r=−0.03 (p=0.67)†
*APOE4* carrier, n (%)¶	54 (30.1)	32 (28.8)	0.69‡	p=0.08*
Childhood cognition, z-score, mean (SD)	0.40 (0.72)	0.48 (0.71)	0.39§	r=0.06 (p=0.92)†
Advanced education, n (%)	99 (56.3)	52 (46.8)	0.15‡	p=0.15*
Non-manual occupation (parental), n (%)	70 (39.8)	43 (38.7)	0.90‡	p=0.83*
Non-manual occupation (own adult), n (%)	22 (12.5)	20 (18.0)	0.23‡	p=0.32*
PTA best hearing ear, dB HL, median (IQR)	17.5 (13.8–21.3)	31.3 (27.5–37.5)	<0.001*	n/a
Hearing aid use, n (%)	3 (1.7)	39 (35.1)	<0.001‡	p<0.001*
Self-reported tinnitus, n (%)	30 (17.1)	36 (32.4)	0.004‡	p=0.0025*

Hearing impairment at baseline was defined as a PTA of greater than 25 Decibels in the best hearing ear.

*Unadjusted Mann-Whitney U test.

†Spearman’s rank correlation.

‡Unadjusted Fisher’s exact test.

§Unadjusted Student’s t-test.

¶Defined on basis of presence of at least one *APOE4* allele there was a small number of *APOE4* homozygotes including 6 (3.4%) with normal hearing and 2 (1.8%) with impaired hearing (p=0.49).

n/a, not available; PTA, pure tone average; TIV, total intracranial volume.

Area under the curve (AUC) analyses were performed to investigate whether hearing impairment or PTA predicted baseline Aβ-status. Generalised linear models using the gamma distribution and log link were used to investigate whether PTA predicted baseline WMHV, as per previous work.^5^

Linear regression models were used to test associations between baseline peripheral hearing ability and longitudinal measurements of brain volume (whole brain, total hippocampal atrophy and ventricular expansion) and cognitive performance following adjustment for baseline Aβ-deposition, baseline WMHV, age, sex, *APOE* genotype, education, childhood cognition and socioeconomic position. For BSI analyses, TIV was included as a covariate. To investigate if associations between hearing and atrophy (BSI) relate to Alzheimer’s or cerebrovascular disease, we assessed interactions with Aβ-deposition and baseline WMHV. We additionally assessed if relationships between atrophy (BSI) and rates of cognitive decline (change in PACC) were influenced by hearing.

Results were expressed using a standard statistical threshold of <0.05, as well as a more conservative Bonferroni-corrected threshold of p<0.0125, based on the four primary outcomes of interest.

## Results

111 out of 287 participants had evidence of hearing impairment. Relationships between hearing and demographic variables are detailed in table 1.

As per cross-sectional analysis,^5^ there was no evidence that hearing ability independently predicted Aβ-status or WMHV at baseline in this longitudinal sample. In this longitudinal sample 22/176 participants with normal peripheral hearing and 26/111 hearing impaired participants were classified as amyloid positive (mean Standardized uptake value (SUVR) 0.55 and 0.57, respectively, using an eroded subcortical white matter reference region). A base model combining age, sex, WMHV, education, childhood cognition, socioeconomic position and *APOE* genotype, provided an AUC for Aβ-positivity of 0.73 (95% CI 0.66 to 0.81) and predictive ability was not significantly improved by inclusion of hearing impairment (AUC 0.76, 95% CI 0.69 to 0.83) or PTA (AUC 0.75, 95% CI 0.68 to 0.82). After adjustment for age, sex, Aβ-status, education, childhood cognition, socioeconomic position and *APOE* the ratio of mean WMHV for hearing impaired: not hearing impaired was 0.83 (95% CI 0.62 to 1.10 p=0.2) and the proportional change in mean WMHV for each dB HL increase in PTA was 0.99 (95% CI 0.98 to 1.00, p=0.5).

**Figure 1:**
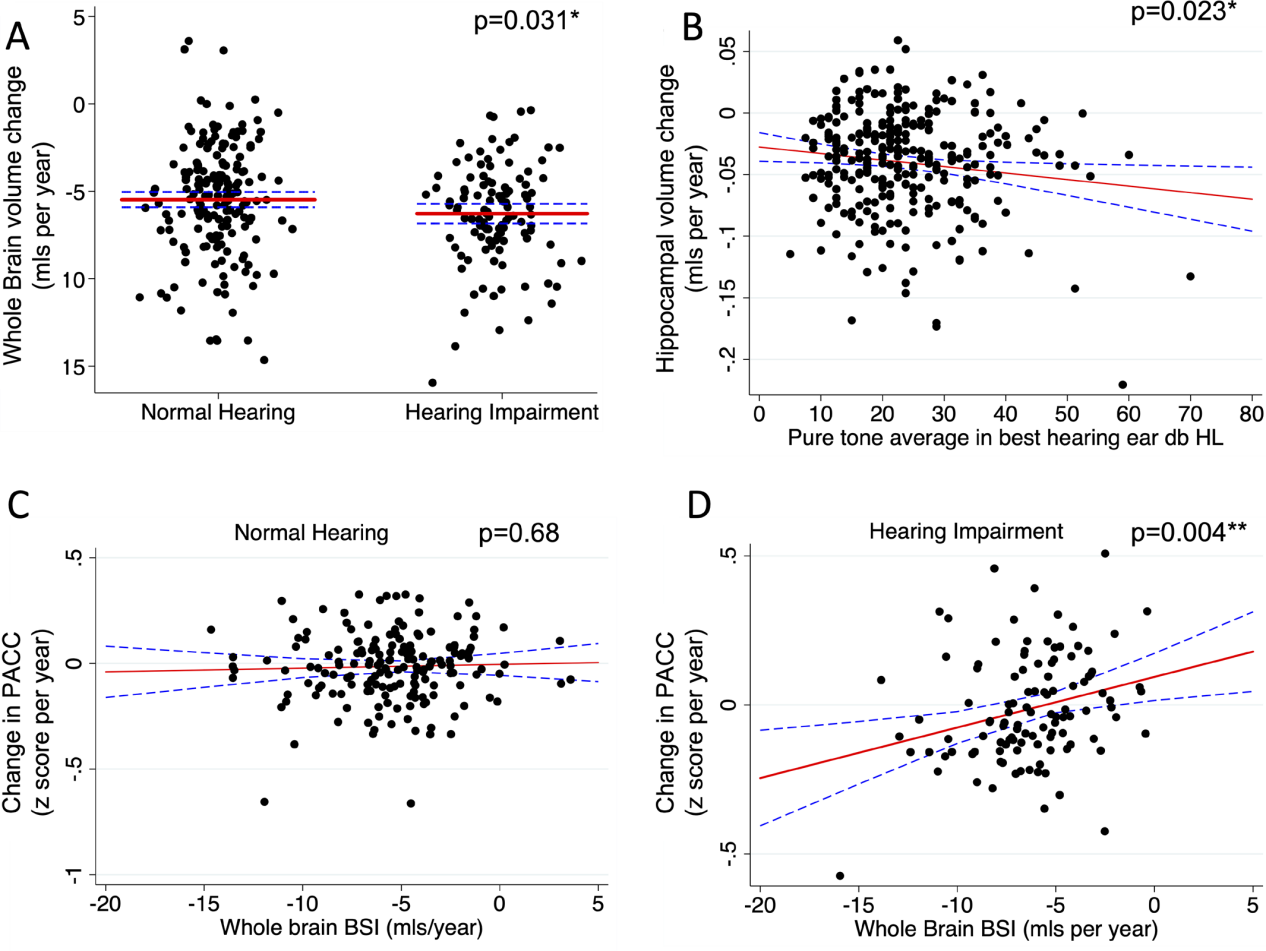
(A) Relationship between hearing impairment and whole brain atrophy rates. (B) Relationship between pure tone average and hippocampal atrophy rates. (C) Relationship between change in cognitive performance and whole brain atrophy in participants with normal hearing. (D) Relationship between change in cognitive performance and whole brain atrophy in participants in participants with peripheral hearing impairment. Scatter plots show the raw data. The solid line represents the marginal adjusted mean following regression modelling (adjusted for baseline amyloid deposition, baseline white matter hyperintensity volume, age, sex, *APOE* genotype, educational attainment, childhood cognitive ability, socioeconomic position and total intracranial volume). The dashed lines represent the 95% CIs. Negative volume change values correspond to increased rates of brain atrophy. *p<0.05; **p<0.0125 (Bonferroni threshold). PACC, Preclinical Alzheimer’s Cognitive Composite.

Compared with those with preserved hearing, there was evidence that hearing impaired individuals had faster rates of whole brain atrophy (p=0.031) (table 2 and figure 1A). There was evidence that higher PTA (worse hearing) also predicted faster rates of hippocampal atrophy (p=0.023) (table 2 and figure 1B). These results were not significant using a Bonferroni-based statistical threshold, but all longitudinal volume results were directionally consistent with each other making it less likely these were a consequence of a type I error. There was no evidence that hearing ability predicted change in PACC score (table 2). These effects remained after adjustment for Aβ-status and WMHV. Furthermore, there was no evidence of an interaction between hearing ability and Aβ-status or WMHV in terms of their effects on atrophy or cognitive change.

**Table 2:** Linear regression models testing associations between baseline hearing ability and cross-sectional/longitudinal measurements of brain volume (whole brain, total hippocampal and ventricular), and the Preclinical Alzheimer’s Cognitive Composite following adjustment for amyloid deposition, white matter hyperintensity volume, age, sex, *APOE* genotype, educational attainment, childhood cognitive ability, socioeconomic position and total intracranial volume (for volumetric brain analyses only)

	β-coefficient (95% CI; p value)	
	Hearing impairment (binary)	PTA (per 1 dB increase)
Baseline whole brain volume (mL)	0.98 (−8.65 to 10.6; 0.84)	−0.02 (−0.51 to 0.48; 0.95)
Whole brain volume change (mL/year)	**−0.80** **(−1.52 to –0.08; 0.031**)	−0.026 (−0.06 to 0.008; 0.13)
Baseline hippocampal volume (mL)	0.11 (−0.02 to 0.24; 0.11)	0.0065 (−0.0051 to 0.0136; 0.069)
Hippocampal volume change (mL/year)	−0.0085 (−0.018 to 0.0012; 0.088)	**−0.00053** **(−0.00098 to –0.00008; 0.023**)
Baseline ventricle volume (mL)	−1.02 (−4.21 to 2.06; p>0.05)	−0.04 (−0.17 to 0.08; >0.05)
Ventricle volume change (mL/year)	0.06 (−0.14 to 0.25; >0.05)	−0.0002 (−0.0073 to 0.0072; >0.05)
PACC baseline (z-score)	−0.067 (−0.202 to 0.067; p=0.33)	−0.005 (−0.011 to 0.001; p=0.12)
PACC change (z-score)	0.003 (−0.037 to 0.043; p=0.88)	−0.002 (−0.0021 to 0.0016; p=0.82)

p<0.05 highlighted in bold.

Ventricle BSI model did not fully meet assumptions for linear regression so bootstrapping (2000 replications) was used to produce bias-corrected and accelerated 95% CIs and meant precise p value calculation was not possible.

BSI, boundary shift integral; PACC, Preclinical Alzheimer’s Cognitive Composite; PTA, pure tone average; SUVR, Standardized uptake value.

There was evidence of an interaction between hearing impairment and the relationship between whole brain atrophy and cognitive change (p=0.031): while there was no evidence of an association between rates of brain atrophy and cognitive change in participants with preserved hearing (β-coefficient=0.002, p=0.68, figure 1C), in those with hearing impairment faster rates of whole brain atrophy predicted greater cognitive change (β-coefficient=0.017, p=0.004, figure 1D). Again, all observed relationships were independent of Aβ-status and WMHV. This relationship remained evident following removal of an outlier with BSI less than −15 mL per year (β-coefficient=0.012, p=0.042).

## Discussion

We demonstrate that peripheral hearing impairment predicts faster rates of brain atrophy in older adults. This is consistent with previous reports,^11 12^ but extends these findings to show these effects are independent of Aβ-status and WMHV suggesting that relationships between hearing loss and neurodegeneration may be driven by mechanisms other than Alzheimer’s or cerebrovascular disease.^7^ This does not, however, preclude the possibility that accelerated atrophy involving key structures such as the hippocampus could prime or accelerate the subsequent emergence of neurodegenerative pathologies such as Alzheimer’s disease.^2^

Hearing impairment did not predict cognitive change in those with normal hearing, but faster rates of whole brain atrophy did predict greater cognitive change in participants with hearing impairment. Hearing loss imposes a cognitive load, particularly on processes that require speech comprehension: our findings suggest that this may cause cognitive dysfunction to become manifest, in situations where brain reserve is already limited (e.g. due to increased underlying brain atrophy).^13^ Future work examining the precise mechanisms that predict cognitive change in the context of hearing impairment are required. In particular, investigating to what extent auditory impairment is a risk factor for, or manifestation of neurodegenerative processes, and whether these can be mitigated by appropriate hearing interventions.^4 14^

It is important to note that the effects of peripheral hearing ability on atrophy rates and cognition in this sample of cognitively healthy older adults were subtle and the clinical meaningfulness of these effects is uncertain. Longer-term follow-up looking at data such as conversion to dementia will be vital to establish the true relevance of this finding.

This study benefits from detailed longitudinal phenotyping as well as a unique level of age-matching. Limitations include the fact that some of the cognitive tests have an auditory component, reduced sample size and a relatively selective population due to participant drop-out, as well as relatively short duration of follow-up.^15^ Future work with larger sample sizes, longer follow-up durations and more detailed biomarker characterisation will be of value. In addition, this study does not investigate central auditory processing, a cognitively demanding process involving a range of brain areas, which has been shown to have particular relevance in neurodegenerative conditions and should be an important focus for future work.^16^

Our data suggest a complex interplay of hearing ability, neurodegeneration and cognition and implicate pathways separate to those typically implicated in Alzheimer’s and cerebrovascular disease.
